# Population genetic structure of *Diaphorina citri* Kuwayama (Hemiptera: Liviidae): host-driven genetic differentiation in China

**DOI:** 10.1038/s41598-018-19533-5

**Published:** 2018-01-24

**Authors:** Lixue Meng, Yongmo Wang, Wen-Hua Wei, Hongyu Zhang

**Affiliations:** 1 Key Laboratory of Horticultural Plant Biology (MOE), State Key Laboratory of Agricultural Microbiology, Institute of Urban and Horticultural Entomology, College of Plant Science and Technology, Huazhong Agricultural University, Wuhan, 430070 China; 2 Hubei Insect Resources Utilization and Sustainable Pest Management Key Laboratory, College of Plant Science and Technology, Huazhong Agricultural University, Wuhan, Hubei P.R. China; 3 Department of Women’s and Children’s Health, Dunedin School of Medicine, University of Otago, Dunedin, 9016 New Zealand

## Abstract

The Asian citrus psyllid *Diaphorina citri* Kuwayama is a major pest in citrus production, transmitting *Candidatus* Liberibacter asiaticus. It has spread widely across eastern and southern China. Unfortunately, little is known about the genetic diversity and population structure of *D. citri*, making pest control difficult. In this study, nine specifically developed SSR markers and three known mitochondrial DNA were used for population genetics study of *D. citri* using 225 samples collected from all 7 distribution regions in China. Based on the SSR data, *D. citri* was found highly diverse with a mean observed heterozygosity of 0.50, and three subgroups were structured by host plant: (i) Shatangju, NF mandarin and Ponkan; (ii) Murraya paniculata and Lemon; (iii) Citrus unshiu, Bingtangcheng, Summer orange and Navel. No significant genetic differences were found with mtDNA data. We suggested the host-associated divergence is likely to have occurred very recently. A unimodal distribution of paired differences, the negative and significant Tajima’s *D* and Fu’s *F*_*S*_ parameters among mtDNA suggested a recent demographic expansion. The extensive citrus cultivation and increased suitable living habitat was recommended as a key for this expansion event.

## Introduction

The Asian citrus psyllid, *Diaphorina citri* Kuwayama (Hemiptera: Liviidae), is the most serious pest of citrus worldwide, primarily due to its role as a vector for “*Candidatus* Liberibacter asiaticus”, the bacterium that causes the highly destructive Asian huanglongbing (HLB) (citrus greening)^1^. HLB affects all citrus cultivars^2,3^. Severely HLB-infected trees often appear stunted, sparsely foliated and die back, losing their economic viability. Ledford^4^ reported that this disease has slashed US orange production in half over the past decade, and threatens to destroy the US$3.3-billion industry entirely. To date there is no effective control technique for this disease^5^, and control of *D. citri* has been one of three critical components of HLB management in addition to planting pathogen-free nursery stock and removing inoculums by destroying infected trees^6^.

*Diaphorina citri* was first described in Taiwan in 1907 and currently has spread throughout much of Asia^6,7^. In Brazil, *D. citri* was found in the 1940s^8^, expanded its range to Florida in the late 1990s^9^, and is now well established in citrus producing regions of the United States^10,11^ as well as Mexico, Belize, Costa Rica, the Caribbean and South America^12^. The wide distribution of *D. citri* and probable high genetic diversity make any suppression efforts challenging. Taking China as an example, after a first found in Taiwan in 1907^6^, *D. citri* spread quickly to the coastal provinces including Guangdong, Fujian and Guangxi, and then to inland provinces such as Hunan, Jiangxi, Yunnan and Sichuan^13^. During the invasion course, *D. citri* has adapted itself to a wide range of geographic environments and host species, becoming ubiquitous in almost all citrus growing areas in China^14,15^.

Deciphering genetic diversity, population structure, as well as history and adaptation processes is essential for developing effective suppression protocols and sustainable management strategies for insect pests^16^. DNA markers such as mtDNA, SSRs, RAPD, AFLP and ESTs have been used as popular marker systems in insect genetics research^17^. In the last few years, much attention has been drawn to phylogeographic studies of *D. citri* populations; most of these studies utilized mitochondrial genes (i.e., the COI gene) as genetic markers. Studies of *D. citri* populations with the COI gene in America suggested two independent founder events occurred in South and North America^18^. Boykin^19^ found two major haplotype groups with preliminary geographic bias between southwestern and southeastern Asia using the COI gene. *Diaphorina citri* was reported to have formed two geographically and genetically structured groups (the eastern versus western side of the state of São Paulo) and has undergone a recent population expansion in Brazil based on COI sequences^20^.

As a marker, some of the unique characteristics of mtDNA compared to nuclear DNA are a high rate of nucleotide substitution, maternal inheritance, and little or no recombination^17,21^. Generally, mtDNA is assumed to evolve under neutral or nearly-neutral selection^21^. However, the Neutral theory is only valid for slow evolving genes yet to reach Maximum Genetic Diversity (MGD) and using the fast evolving part is wrong to analyze phylogy^22^. Besides, the use of only mtDNA for phylogeographic and population genetic studies is questioned, because within-species diversity does not reflect population size or ecology, and it is difficult to investigate evolutionary questions^23^. Utilizing both slow evolving and fast evolving molecular markers together can generate a comprehensive picture of both ancient and recent population histories and thus becomes necessary ^24,25^. Owing to their rapid evolution, extensive genome distribution and high level of polymorphism, as well as apparent neutrality and co-dominant behavior, Short Simple Repeats (SSR) or microsatellite markers have become a useful marker in studies of ecology, evolution, conservation, and population biology of many insect species^26,27^. Compared to the AFLP system, SSR markers are also sufficiently abundant to generate large numbers of markers^17^ and have been widely used in plants, animals, and microorganisms^28–32^. The joint application of mtDNA and SSR markers in the same population will make the results of phylogeography and population genetics analyses more validated and additional genetic information will be gained^22^. To date, no study has been undertaken in *D. citri* to investigate the genetic diversity and relationships among populations using different classes of genetic markers simultaneously on the same samples. And, no researcher has tested if mtDNA genes are neutral before using them.

This study aimed at (1) investigating the genetic diversity of *D. citri* populations in China using three mtDNA genes (COI, Cytb and ND5) and nine newly developed SSR markers; (2) investigating the factors shaping the population genetic structure of *D. citri* populations; and (3) investigating the demographic history of *D. citri* populations in China to propose a hypothesis on the expansion of this pest under China conditions. Through the study, we wish to provide a foundation to improve the monitoring and control of *D. citri*.

## Results

### SSR markers developed

In *D. citri*, 144 SSR loci were obtained after scanning the whole genome with SSR Hunter 1.3. These motifs contained dinucleotide (57.6%) or trinucleotide (38.9%) motifs. The others were less frequent (9.4% tetranucleotide, 1.4% pentanucleotide). Of the 144 potentially amplifiable loci, 50 primer pairs were tested and 20 were selected according to their amplification. Thirteen primer pairs were validated on a panel of samples, i.e., production of specific PCR products of the expected size, and tested for polymorphism. Nine of these 13 loci (69.2%) proved to be polymorphic for all individuals (Table 1). These loci contained 7 dinucleotide and 2 trinucleotide motifs.

**Table 1:** Characteristics of nine polymorphic SSR primers developed in *D. citri*.

Locus ID	Primer sequences (5′− 3′)	Repeat motif	Size range (bp)^a^	Ta (°C)	Accession no.	*A*	*Ho*	*H*_*E*_	*F*_*IS*_
Dci-1	F:GTTGGGAATAGGCCTGACAA R:ATCAGGACGGTGGACACTTT	(TC)_22_	166–218	61	KY053427	6	0.636	0.483	−0.331
Dci-7	F:GATGGAAGAGAGAAAAGGGAGA R:GGGTGGGGTCATTCAAAATA	(AG)_20_	217–339	60	KY053428	13	0.638	0.798	0.187
Dci-8	F:TAACCATCGTCAGGCAATCA R:TGGGAGGGAAAGATTGAGAA	(TC)_15_	250–276	62	KY053429	12	0.197	0.837	**0.763**
Dci-22	F:GCTTCTTGTCGCTTGTGC R:GAGTTTGGTGGATGTGCC	(GA)_27_	128–216	60	KY053430	18	0.915	0.904	−0.015
Dci-26	F:TTAGTGGAGATGCCTGAT R:TCGCCAACATACGAAACG	(TTC)_25_	104–110	60	KY053431	3	0.877	0.571	−0.546
Dci-29	F:CAATCTTGATACTGTGGGTT R:TGGAAGTGAGGGTGAGAA	(TTC)_22_	184–262	60	KY053432	12	0.192	0.317	**0.387**
Dci-33	F: TGCTTACTTTTGTAGACC R:ATACCTCCATAACATCCC	(GA)_8_	125–159	60	KY053433	13	0.102	0.234	**0.560**
Dci-34	F:CCTGGGATAATGGATGAC R:CTGTTGTACCCTGCTTCG	(GT)_7_	104–122	60	KY053434	11	0.442	0.442	−0.016
Dci-36	F:TATCAATCCTACCAAAGT R:TGAAGGGGCATTACAACA	(GA)_9_	189–209	60	KY053435	11	0.479	0.727	**0.338**

*A*, number of alleles detected; Ta, annealing temperature using the genotyping protocol of Schuelke^114^. *H*_O_, observed heterozygosity; *H*_*E*_, expected heterozygosity; ^a^the allele sizes of the PCR products including the length of the fluorescent-labeling M13 (−21) sequencing primer (18 bp); *F*_*IS*_, inbreeding coefficient; *Significant deviation from Hardy-Weinberg equilibrium (P < 0.05).

All loci showed no evidence of fragment-length homoplasy. A few single nucleotide polymorphisms (SNPs) were identified sporadically in the flanking regions of SSRs in the makers Dci-22 and Dci-26. Since SNPs were not located in the target region of primers and had no affect on the length of amplicons, they would not hamper the amplification of the markers or data analysis^33^. Therefore, all nine SSRs were used for further population genetics study.

### Overall genetic variation

Different parameters of genetic variation were estimated. The average number of alleles of the identified SSR loci was 11 (ranging from 3 to 18) with mean heterozygosity (*H*_*O*_) of 0.484 ranging from 0.100 (locus Dci-33) to 0.931 (locus Dci-22) (Table 1). Tests of linkage disequilibrium (LD) between each pair of loci using GENEPOP v3.1 across all populations found no significant linkage suggesting that these SSR loci are independent from each other (*p* = 0.391). Four of nine loci were found to have deviated from HWE (*p* < 0.05) after Bonferroni correction in all populations. Further tests (i.e., *F*_*IS*_) revealed that the deviations from HWE may be caused by heterozygote deficiency (Table 1). MICROCHECKER found some evidence of null alleles but each with a low frequency less than 0.081, meaning that population substructure or selection upon the loci may be responsible for the observed HWE deviations. The variation parameters obtained in our work are mostly above the range of those reported SSR for *D. citri* in previous works^34,35^, suggesting a high variation level in the 9 SSR loci studied.

We sequenced three mitochondrial fragments from 225 *D. citri* individuals. A total of 2398 bp in a concatenated sequence from COI, Cytb and ND5 were analyzed. There were 72 (3%) variable positions in the concatenated sequence (COI had 2.56%, Cytb had 3.34% and ND5 had 3.07%) with a transition: trans-version ratio of 1:1. In total, 26 *D. citri* haplotypes were identified from the concatenated mtDNA in which all populations had low diversity at both the nucleotide (π: 0.00082) and haplotype (Hd: 0.4957) levels.

### Phylogeny derived from SSR and mtDNA data

**Figure 1:**
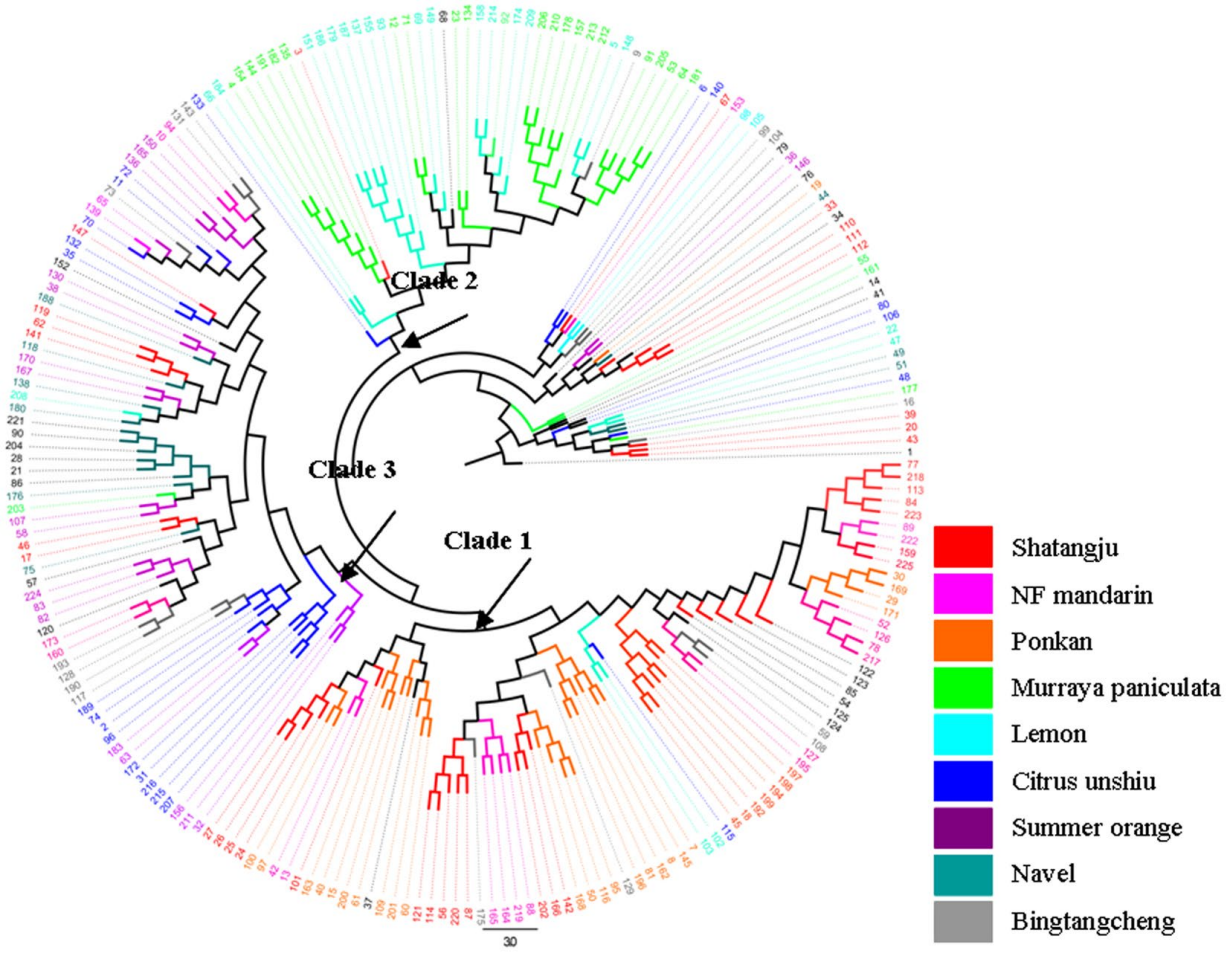
Neighbor-joining tree constructed using pairwise differences for *D. citri* individuals. The populations are color-coded according to the pre-defined ancestry categories.

The unrooted NJ phylogram generated from the SSR data indicated a high degree of clustering by host plant species (Fig. 1). Samples from the nine host plants formed three main discrete clusters. *Diaphorina citri* individuals from Shatangju, NF mandarin and Ponkan were clustered in Clade 1; those from Murraya paniculata and Lemon in Clade 2; those from Citrus unshiu, Bingtangcheng, Summer orange, and Navel in Clade 3. There is no evidence of clustering by geographic location suggesting that the phylogenic lineages were mainly associated with the type of host plant regardless of sampling location^36^.

Saturation analysis results, conducted using DAMBE v.5.3.8^37^, indicate no significant saturation for all three codon positions (P < 0.0001; Iss values <Iss.c values). Neither the single gene nor concatenated data sets showed any sign of pervasive homoplasy (CI > 0.5).

Parsimonious analysis of the concatenated mtDNA sequence yielded three equally parsimonious trees, from which a strict consensus tree was constructed. The topology of consensus tree is presented as an unrooted network with scaled branches (data not shown).

Based on the 26 identified *D. citri* mtDNA haplotype sequences, branch lengths in terms of both synonymous (syn) and non-synonymous (nonsyn) sites for each mitochondrial gene were estimated and shown in Fig. S1. The mtDNA diversification as defined by at nonsyn sites exhibited few hierarchical structures (Fig. S1). At syn sites, all genes almost showed no divergence across the entire tree. There was no evidence of phylogenetic structuring by either host plant or sampling location which is inconsistent with the NJ tree from SSR data.

### Genetic diversity and population structure based on SSR data

The AMOVA analysis result revealed over 80% variance came from individuals within population which indicated that great genetic variation existing within the populations. The partitioning of total genetic variation in three host plant groups using AMOVA indicated 10.83% genetic diversity among groups, 4.78% among host plant populations (Table 2). AMOVA also revealed significant F-statistics for each hierarchical level (Table 2), suggesting that *D. citri* has strong and significant genetic differentiation at the host plant level. In contrast, AMOVA detected 7.83% of variation among locations populations and 92.17% within locations populations (Table 2), hinting very low genetic differentiation at the location level.

**Table 2:** AMOVA in *D. citri* populations categorized by either host plant or location and corresponding fixation indices based on SSR markers.

Source of variation	df	SS	VC	%V	F	P
SSR analysis						
Among 3 host plants groups						
Among groups	2	417.24	0.312	10.83	F_CT_ = 0.108	0.001
Among populations within groups	7	206.06	0.155	4.784	F_SC_ = 0.063	0.004
Within populations	752	5059.14	2.413	84.41	F_ST_ = 0.025	0.004
Among 7 locations populations						
Among populations	6	323.809	0.213	7.83	F_SC_ = 0.041	0.027
within populations	1790	4418.753	0.246	92.17	F_ST_ = 0.052	0.034
Concatenated mtDNA analysis						
Among 3 host plants groups						
Among groups	2	10.88	0.011	3.49	F_CT_ = 0.034	0.101
Among populations within groups	74	33.07	0.034	41.12	F_SC_ = 0.011	0.055
Within populations	178	57.99	0.051	55.39	F_ST_ = 0.005	0.215
Among 7 locations populations						
Among populations	6	60.43	0.045	58.54	F_SC_ = 0.045	0.005
within populations	199	46.49	0.034	41.46	F_ST_ = 0.034	0.104

df, Degrees of freedom; SS, sum of squares; VC, variance components; %V, percentage of variation; F, multilocus F-statistic; F_CT_, Variation among groups; F_SC_, Variation among populations within groups; F_ST_, variation within population.

**Figure 2:**
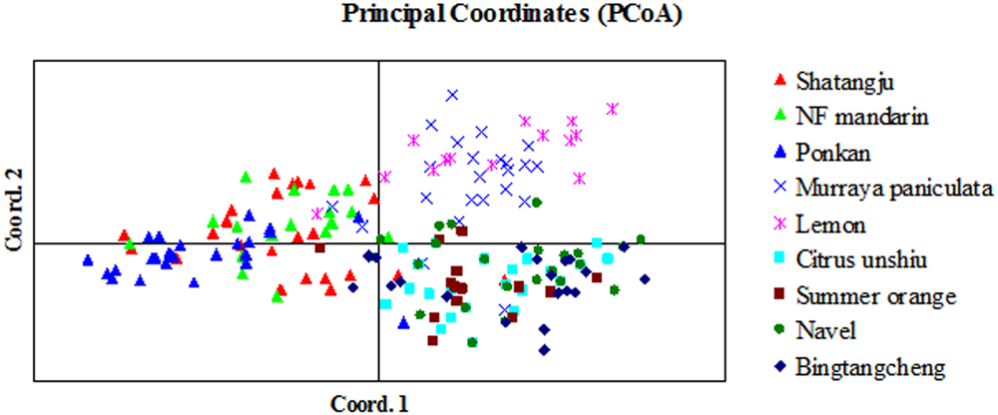
Principal coordinate analysis (PCoA) of pairwise distances between individuals of *D. citri* with SSR markers. Host plant populations are indicated by different colors.

Principal coordinate analyses showed that the first two components jointly explained 75.6% of the genetic variation of the *D. citri* populations (right panels, Fig. 2). The first component (bottom right, Fig. 2) separated *D. citri* on Citrus unshiu, Bingtangcheng, Summer orange and Navel from the remaining, whereas the second component (top right, Fig. 2) separated *D. citri* on Murraya paniculata and Lemon from the rest of the remaining (Fig. S2). PCoA analyses based on sampling location found no clear structure or differences (Fig. S3).

The *D. citri* populations were grouped according to their host plants. Estimates were made of the genetic diversity present among host plant populations. The expected heterozygosity (*H*_*E*_) in the overall *D. citri* populations’ data set was 0.523, with an overall observed heterozygosity (*H*_*O*_) of 0.492. Four host plant populations (Citrus unshiu, Summer Orange, Navel and Bingtangcheng) showed higher observed heterozygosity than expected; although in many cases this difference was not significant (Table 3). Mean *H*_*E*_ values ranged from 0.432 (Ponkan) to 0.591 (Murraya paniculata), and mean *H*_*O*_ ranged from 0.377(NF mandarin) to 0.617(Summer Orange) (Table 3). The percentage of polymorphic loci per host plant population ranged from 88.9% to 100% (Table 3).

**Table 3:** Genetic diversity indices of *D. citri* from different host plants based on SSR markers.

**Population**	***N***	***N***_***A***_	***H***_***O***_	***H***_***E***_	***Nei’s***	***Np***	***P*** **(%)**
Shatangju	32	53	0.425 ± 0.365	0.494 ± 0.294	0.486 ± 0.288	9	100
NF mandarin	22	51	0.377 ± 0.347	0.453 ± 0.277	0.441 ± 0.270	8	100
Ponkan	37	42	0.404 ± 0.371	0.432 ± 0.274	0.415 ± 0.270	8	88.89
Murraya paniculata	25	56	0.391 ± 0.272	0.591 ± 0.207	0.580 ± 0.203	9	100
Lemon	22	51	0.498 ± 0.248	0.587 ± 0.241	0.573 ± 0.235	9	100
Citrus unshiu	22	46	0.550 ± 0.323	0.540 ± 0.271	0.526 ± 0.263	9	100
Summer Orange	22	41	0.617 ± 0.408	0.510 ± 0.280	0.498 ± 0.273	8	88.89
Navel	23	41	0.578 ± 0.356	0.548 ± 0.235	0.536 ± 0.230	9	100
Bingtangcheng	20	39	0.595 ± 0.317	0.556 ± 0.218	0.542 ± 0.212	9	100

**Figure 3:**
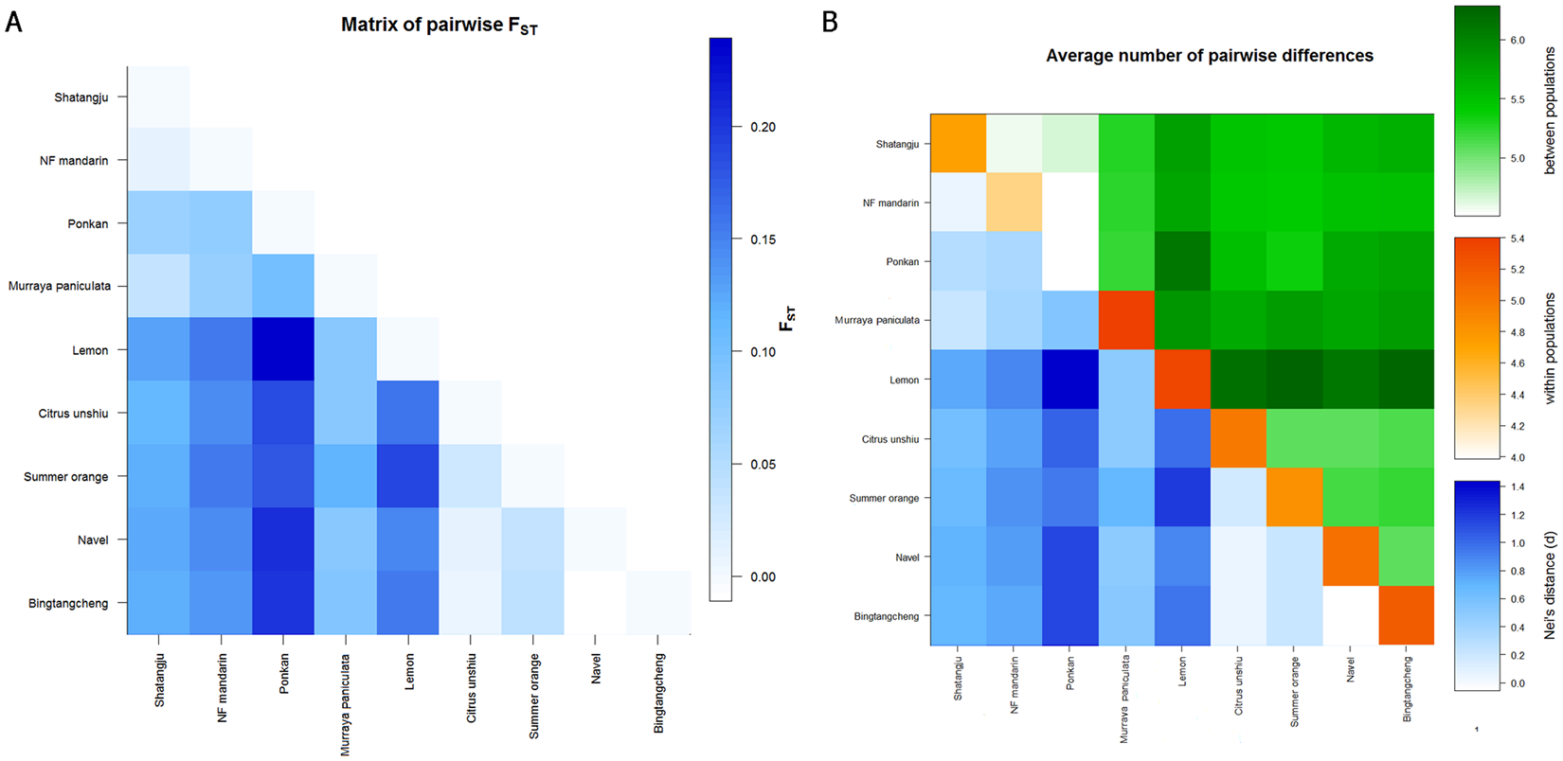
Graphic representation of *D. citri* host plant population relationships as described by average number of a matrix of pairwise F_ST_ values and pairwise differences (π). (**A**) Population relatedness represented by pairwise F_ST_ (<0.1: minimal genetic differentiation; between 0.1 and 0.2: moderate genetic differentiation; >0.2 high differentiation); (**B**) Population differences measured by pairwise differences π (within population on the diagonal, color coded from orange to red; between populations above the diagonal, color coded green) and genetic distance between populations (below the diagonal, blue).

*N*, Number of samples; *N*_*A*_, Observed number of alleles; *Nei’s*, Nei’s gene diversity; *H*_*O*_, Observed heterozygosity; *H*_*E*_, Expected heterozygosity; *Np*, Number of polymorphic loci; *P*, Percentage of polymorphic loci;

The relationship among the nine host plant populations was further examined using the genetic distances as shown in Table 4. The overall F_ST_ among all host plant populations of *D. citri* was equal to 0.13, significantly different than zero (*p* = 0.01). Pairwise F_ST_ values ranged from 0.001 (Navel to Bingtangcheng) to 0.398 (Ponkan to Lemon) (Fig. 3A and Table 4), and comparisons of Ponkan to the other and Lemon to the other were higher than any host plant population comparison.

**Table 4:** Nei’s genetic distance (below diagonal) between nine host plant populations of *D. citri*.

**Population**	Shatangju	NF mandarin	Ponkan	Murraya paniculata	Lemon	Citrus unshiu	Summer orange	Navel	Bingtangcheng
Shatangju									
NF mandarin	0.053								
Ponkan	0.019	0.066							
Murraya paniculata	0.230	0.096	0.236						
Lemon	0.069	0.129	0.398	0.158					
Citrus unshiu	0.192	0.251	0.218	0.157	0.373				
Summer orange	0.198	0.276	0.227	0.211	0.330	0.065			
Navel	0.224	0.317	0.225	0.171	0.324	0.090	0.023		
Bingtangcheng	0.220	0.312	0.220	0.157	0.286	0.076	0.022	0.001

We also generated estimates of the variance within vs. between host plant populations using pairwise differences (π) as a measure of population subdivision (Fig. 3B). Each of these populations showed within population heterogeneity (Fig. 3B). High pairwise differences were found between the host plant populations indicating genetic differentiation was present among *D. citri* (color coded green in Fig. 3B).

### Genetic diversity and population structure based on mtDNA

Nucleotide (π) and haplotype (Hd) diversity levels calculated with syn sites was all lower than nonsyn sites as shown in Fig. S4, this is because syn sites are fast evolving and more responding to fast changing environmental factors and hence under selection. Nonsyn sites may be slow evolving and under less selective pressure. Therefore, the following analyses were all based on nonsyn sites in the concatenated mtDNA.

**Figure 4:**
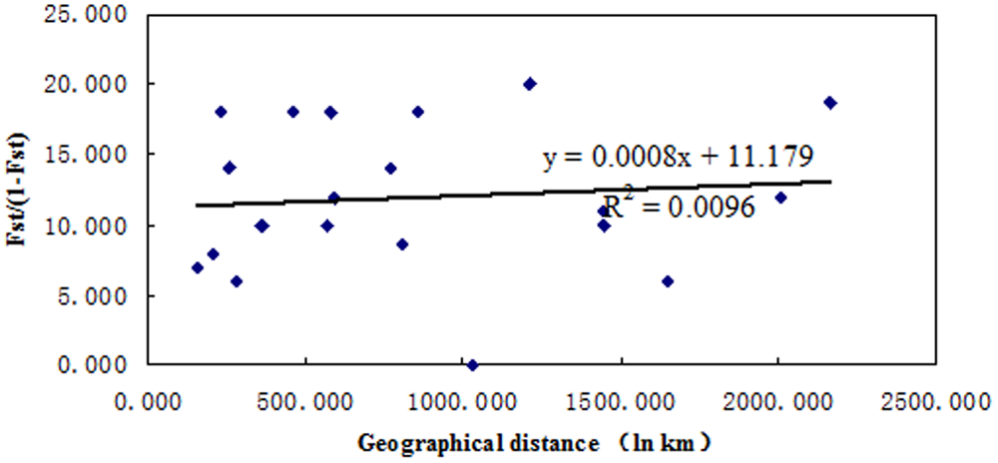
Scatter plots of genetic distance vs. geographical distance for pairwise population comparisons (r^2^ < 0.01, *p* ≥ 0.16; 10 000 randomizations).

Resultant tree based on the concatenated mtDNA sequence did not show any significant spatial structuring due to host plant species or sampling location.The AMOVA of the concatenated mtDNA analysis revealed that 41.46% of genetic variation could be explained by the variation within populations, whereas the remaining (58.54%) came from variation among populations. Further Mantel tests of isolation by distance revealed no significant relationship between geographical and genetic distances (r^2^ < 0.01, *p* ≥ 0.32) (Fig. 4). No population structure in slow sites in mtDNA may be consistent with the fact that this species in China has only a relative short history.

**Figure 5:**
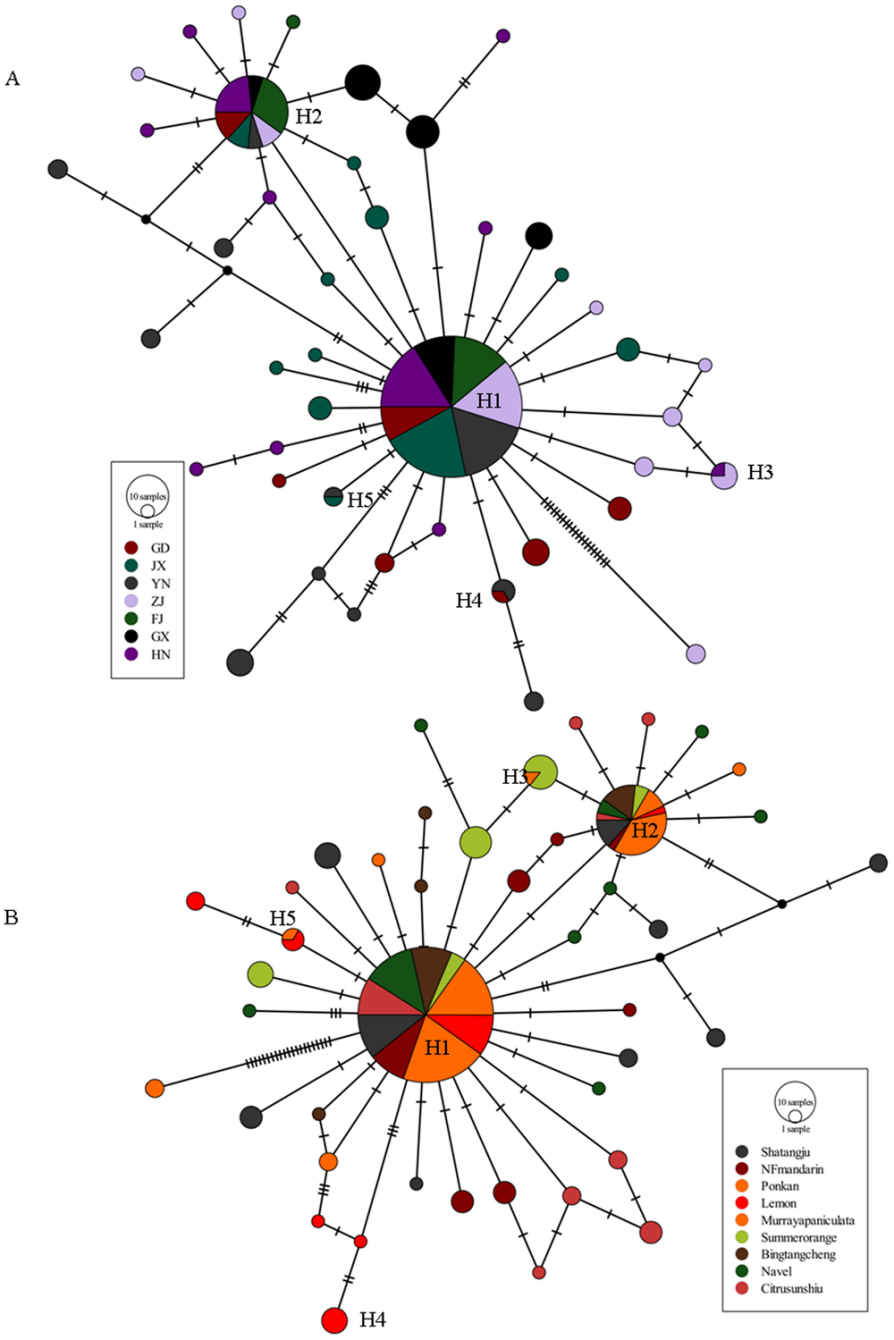
Median-joining networks constructed with the concatenated mitochondrial sequences, represented by (**A**) sampling location and (**B**) host plant. Circle sizes are proportional to the haplotype frequency. Distances are proportional to mutation number between haplotypes.

The strongest association of haplotype diversity with sampling location was seen in Yunnan province (Hd: 0.62299) (Table 5 and Fig. 5A). Yunnan is a region with diverse climatic types in China and thus could be an ideal refuge for old and new *D. citri* haplotypes. The lowest haplotype diversity (Hd: 0.13333) was detected in Fujian and Guangdong provinces, which seems to contradict the fact that they are among the earliest invaded regions in China. The significantly low diversity level may be due to excessive pesticide use and removal of HLB-infected trees for pest control, which caused a higher citrus distribution disturbance and lower overall *D. citri* richness^38^ (Table 1). Jiangxi province is a newly invaded *D. citri* area with high haplotype diversity (Hd: 0.61134), possibly hinting at multiple invasive events or that intra- and/or inter-specific hybridization exists^39^ (Table 5). The strongest association of haplotype diversity with host species was seen in NF mandarin, Lemon and Summer orange sampled from Jiangxi, Yunnan, and Guangxi provinces, respectively, which is consistent with the location association analysis results.

**Table 5:** Summary of nucleotide polymorphism and neutrality test parameters of *D*. *citri* populations.

**Pop ID**	S	Nh	Hd	π	*D*	*F*_*S*_	*R* ^2^
**Total Data**	52	26	0.4943	0.00068	−2.61**	−5.23**	0.0139**
Host plant							
Shatangju	8	6	0.4113	0.0006	***	***	***
NF mandarin	3	4	0.6623	0.0005	***	***	***
Ponkan	22	3	0.1630	0.0012	***	***	***
Murraya paniculata	3	4	0.3477	0.0003	***	***	***
Lemon	8	5	0.6753	0.0017	***	***	***
Citrus unshiu	3	4	0.4503	0.0003	***	***	***
Summer orange	2	3	0.6381	0.0006	***	***	***
Navel	5	5	0.0003	0.0006	***	***	***
Bingtangcheng	2	2	0.1895	0.0003	***	***	***
Location							
Guangdong	1	2	0.1333	0.0001	−0.5290	0.4045	0.1777
Guangxi	2	3	0.5952	0.0005	0.03060	−0.2514	0.1544
Yunnan	9	6	0.6230	0.0014	−0.4702	0.0975	0.0781
Fujian	1	2	0.1333	0.0001	−1.7490	−1.3822	0.1588
Jiangxi	10	9	0.6113	0.0007	−1.1595	−1.5427	0.0730
Hunan	8	8	0.3778	0.0003	−2.6081**	−2.0698	0.0499
Zhejiang	24	5	0.3810	0.0012	−2.5209**	−4.4112*	0.0585*

S, number of segregating sites; Nh, Number of haplotypes; Hd, Haplotype diversity; *D* and/*Fs*, Tajima’s *D* test and Fu’s *Fs*, respectively; R^2^, Ramos-Onsins and Rozas’ R^2^ test; *p < 0.05; **p < 0.001.

### Haplotype network based on nonsyn sites

Based on the above results, we further assessed the associations of 26 unique haplotypes (Fig. 5). The overall pattern was star-like, dominated by the most common and ancient haplotypes (H1, H2), surrounded by the recent haplotypes. The star-like network suggested that most haplotypes differed from H1 and H2 by only a few mutations. The network suggested that *D. citri* experienced population expansion events. The haplotype networks, together with the haplotype distribution information (Fig. 5), indicated the ancestral haplotypes (i.e., H1 and H2) were widespread in the distribution range of *D. citri*, suggesting that a large gene pool was historically present and adequate gene exchange existed among the locations and host plants (Fig. 5).

The *D. citri* population of Yunnan (YN) had a relatively high proportion (80%) of shared haplotypes (H1, H2, H3, H4 and H5), whereas other populations (HN, GD, HN, FJ, ZJ and JX) had 60% shared haplotypes. This would suggest that the *D. citri* population from Yunnan might have established relatively early in these areas. More haplotypes (Nh) were found in Jiangxi (JX) and Hunan (HN), suggesting the existence of more relatively suitable habitats in those two regions (Table 5).

In combined analysis with host species, we observed that Murraya paniculata had a relatively high proportion (80%) of shared haplotypes (H1, H2, H3, H4 and H5) suggesting that Murraya paniculata, which is a widespread plant, is a much preferred host plant for *D. citri*. In many regions of tropical China (Yunnan, Guangxi), pruned hedges of Murraya paniculata are flushing all the year round, which will constitute an extreme psylla reservoir for *D. citri*^40^. This would be the reason why the high haplotype frequency of *D*. *citri* occurred in Murraya paniculata.

### Demographic history

**Figure 6:**
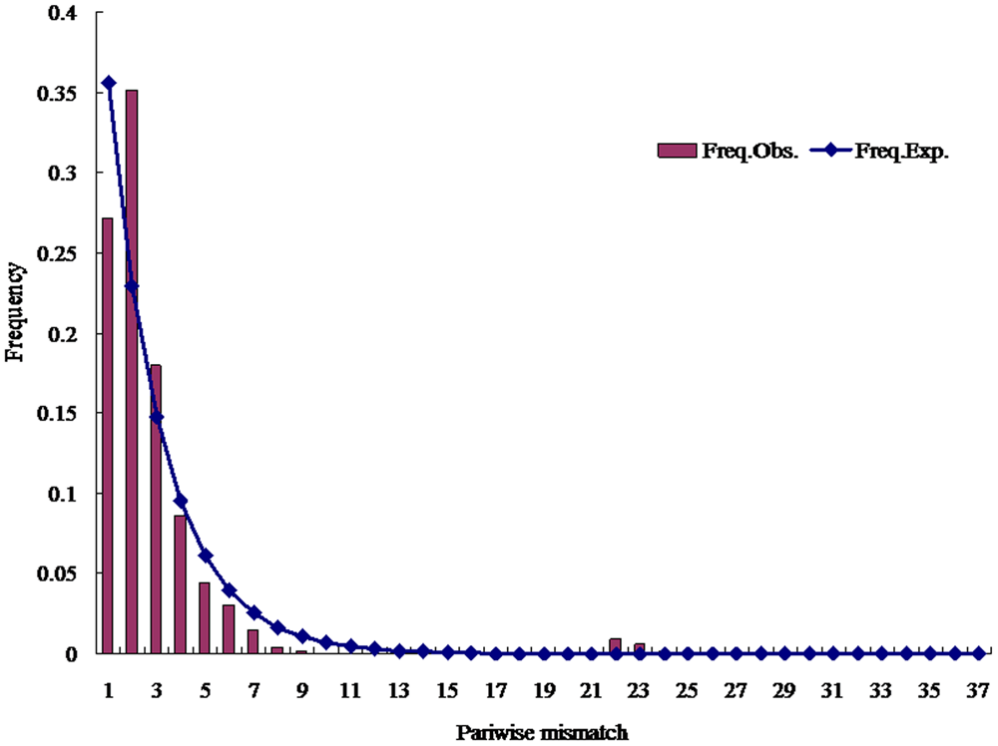
Distribution of pairwise nucleotide differences (mismatch distribution) of the concatenated genes in *D. citri* populations.

Population demographic history was reconstructed using three neutrality tests and mismatch distribution analysis from nonsyn sites. Results from neutrality tests showed significant negative values for Tajima’s *D* and Fu’s *Fs* when all individuals were tested as a single group (Table 5). The results showed a clear deviation from neutrality (*D* and *Fs*) and were further confirmed by the Ramos-Onsins and Rozas’ R^2^ test (Table 5). This test gave significant results, rejecting the null hypothesis of constant size and supporting a recent demographic expansion. The unimodal mismatch distribution obtained for *D. citri* populations (Fig. 6) also suggests *D. citri* in China underwent a process demographic expansion. Statistical analysis of the mismatch distribution supports the model of demographic (SSD = 0.001; P = 0.324) expansion. Moreover, the values of *D. citri* populations size before (θ_0_ = 0.002) and after (θ_1_ = 12.415) the demographic expansion of the populations were calculated.

Also, the applied neutrality tests revealed significant deviations from neutrality for Zhejiang population (Table 5), suggesting that *D. citri* in Zhejiang province have experienced a recent expansion event.

## Discussion

Overall, our present study explores nine polymorphic SSR loci and demonstrates that host-associated differentiation exists among *D. citri* populations in China. This result is derived from the SSR marker analyses, while the mtDNA sequences did not detect any significant genetic differentiation for *D. citri* populations. Furthermore, our results suggest recent population expansions occurred for *D. citri* populations. These results provide new perspectives for *D. citri* control, specifically with respect to host-associated structure among *D. citri* populations.

### Molecular marker resource for *D. citri*

Even though *D. citri* is the most invasive species on citrus^41,42^, there were only a small number of molecular markers (i.e., COI gene and 33 SSR loci) available to study its genetic diversity and population structure^34,35^.

The SSR primer pairs designed in this study from *D. citri* genome sequence information^43^ successfully screened nine polymorphic SSR loci, which were sufficiently variable to allow detailed studies of genetic diversity, LD and population structure. After sequential Bonferroni adjustment^44^, three loci (Dci-8, Dci-29 and Dci-33) in the Chinese *D. citri* populations significantly deviated from HWE (*p* < 0.05), most likely due to heterozygote deficiency or population structure within the individuals. These newly developed markers are likely to be a valuable resource for further population genetic studies of *D. citri* from China and other countries, which could facilitate the conservation and management of *D. citri*.

### Genetic structure of *D. citri* populations

There have been other studies on the genetic diversity of *D. citr* with mtDNA like COI gene. De León *et al*.^18^ identified 23 haplotypes from populations collected in America, while the worldwide haplotype diversity of *D. citri* reported by Boykin *et al*.^19^ is even lower (only 8 haplotypes). In Brazil, Guidolin *et al*.^20^ detected 47 haplotypes using an almost complete sequence of COI gene, indicting a high haplotype diversity (H = 0.839) but still low in nucleotide diversity (π = 0.002). The genetic diversity and tree topology calculated further with nonsyn and syn sites in mtDNA showed a clear pattern of more variation distributed in nonsyn sites (slow evolving gene). Seemingly, neutral processes played a minor role in syn sites (fast evolving gene). Based on MGD thoery, we used nonsyn sites for *D. citri* phylogenic analysis. Genealogy indicated a limited intraspecific variation in *D. citri* population from China showing no clear grouping cluster. Different from mtDNA, the results from the SSR markers for the same *D. citri* populations showed the presence of three host-associated genetically distinct clusters from tree topology and PCoA analysis, which were not related to geographic distance among the *D. citri* populations. Those SSR data suggested that the populations of *D. citri* may be experiencing some degree of host plant-associated differentiation. Nevertheless, the phylogenetic trees constructed from the SSR data could be biased towards host plant selection because the SSR markers were derived mostly from fast evolving sites. Cautions are therefore recommended when interpreting the phylogenetic analysis results^45^.

Such discrepancies between different types of markers are also reported in *Procambarus Clarkii, Syngnathus typhl*e and *Clupea harengus* population genetic studies^46–48^. The inconsistent results obtained from the two molecular markers are generally explained by the features of the two kinds of markers. SSRs, with their high mutation rate of 10^−2^ to 10^−6^/locus/generation^49,50^, can increase the overall resolution to detect genetic differentiation and give clues about recent and contemporary events in the population^51^. By comparison, the slower mutation rate of the mtDNA sequence marker may provide more of a historic than a recent picture of gene flow^52^. On the other hand, such results are consistent with the Maximum Genetic Diversity (MGD) hypothesis^22,36,53^. SSRs as the fast evolving sequences are at saturation genetic diversity (rather than neutral) that is maintained by stabilizing natural selection^36,53^. In our case, host plants provide the selection force. Sequences not compatible with living in a particular plant species would be selected out. Slow evolving sites (mtDNA) are too slow to meet adaptive needs and hence appear neutral within short time frames^53^.

### Host-associated selection driving population genetic differentiation

In this study, *D. citri* populations were found to be structured into three subgroups by host plant: (i) Shatangju, NF mandarin and Ponkan; (ii) Murraya paniculata, Lemon; (iii) Citrus unshiu, Bingtangcheng, Summer orange, and Navel. Mechanisms that may contribute to host-associated differentiation of *D. citri* populations were explored based on the taxonomic, volatile and phenological differences among hosts.

Numerous classification systems of Citrus have been formulated, among which that of Swingle^54^, who described 16 species in Citrus, has been the most widely accepted^55^. Shatangju, NF mandarin, Ponkan and Citrus unshiu all belong to *Citrus reticulate*, while Lemon belongs to *Citrus medical*; Bingtangcheng and Summer orange belong to *Citrus sinensis*. We noticed that the Citrus classification is in accordance with the genetic structure of *D. citri* populations besides Citrus unshiu. Plants are the site of assembly for *D. citri*. Population fluctuations of *D. citri* on citrus and other plants are closely correlated with the rhythm, quantity, and nutritional quality of plant flush because eggs and nymphs are exclusively associated with new growth^56^. Vassiliou^57,58^ found that Lemon (*Citrus medical*) trees flush sporadically throughout the year, providing an important feeding source and refuge for *D. citri* over winter. Murraya paniculata has a more cold-hardy bud break, advancing flushing by six weeks^38^. When compared to *Citrus sinensis*, *Citrus reticulate* trees are more winter-hardy, letting the autumn plant flush extend to November^59^. The prolonged flushing stage would extend the lifespan of *D. citri*. Furthermore, whether or not host plants are available at the early spring and the whole winter poses another high selection pressure on insect populations through effects on diapause^60,61^. Variation in diapauses would contribute to the divergence of host-associated populations of insects^62,63^, as reported in *Rhagoletis pomonella*^64^ and *Diatraea saccharalis*^61^. The flushing characteristics of Lemon and Murraya paniculata described above could make diapauses termination be more beneficial to their *D*. *citri* populations than populations on other citrus species. Therefore, we suggest that host plant phenological differences may contribute to host-associated differentiation in *D. citri* populations.

Host plants provide insects not only nutrients but also secondary substances such as volatiles influencing insect behavior. Chemical and nutritional differences existing among host plants may be a reason for *D. citri* host-associated differentiation. Ehrlich & Raven^65^ found chemical similarities among unrelated food plants used by related butterflies, and suggested that secondary plant substances played the leading role in determining patterns of utilization. Aroma compounds are important for citrus; each cultivar type generates a different profile of volatiles that contributes to its distinct aroma attributes^66^. These volatiles provide a critical clue for host plant selection and orientation in *D. citri*. Linalool was reported to be a major constituent among leaf volatiles in young leaves of most cultivars^67^. γ-terpinene was a major component in leaf oil of *Citrus sinensis*^68,69^ and thymol ethyl ether was only found in leaf volatiles of *Citrus reticulate*^70,71^. Limonene was predominantly found in *Citrus medical* (lemon), and β-bisabolene was only identified in *Citrus medical* leaves^72^. Interestingly, β-bisabolene was also detected in leaf volatiles of Murraya paniculata oil^73^. Therefore, such chemical differences among *D. citri* host plant species could act as an internal driver of locally adapted populations as reported by Ehrlich^65^.

Insects can develop an oviposition preference for the natal plant^63,74^, and assortative mating could occur between individuals on the same host plant^75^. Several lines of evidence have suggested that female host preferences exist in *D. citri*^76^. Investigation of the location and preference of adult *D. citri* on Murraya paniculata, Lemon (*Citrus medical*) and Ponkan (*Citrus reticulate*) found no difference between adult numbers on Murraya paniculata and Lemon, but significantly fewer on Ponkan, suggesting that Murraya paniculata and Lemon are the preferred hosts^77^. Field observations showed that *Citrus medical* suffered the heaviest from *D. citri*, followed by *Citrus sinensis*, with *Citrus reticulate* the lightest^78,79^. Female *D. citri* orient to their plant of origin; males orient to the odor of females and co-specific excretions^80^. This behavior in combination with female oviposition preference behavior likely results in mate choice and limits gene flow between *D. citri* from different host plant clusters^81,82^. In addition, micro-ecological variables such as symbiont composition among different host plants of *D. citri* may also be an important source of host plant-associated natural selection. Study of the population structure and symbiont community of host-adapted *Acyrthosiphon pisum* populations showed that the presence of six facultative (secondary) bacterial endosymbionts was nonrandom distributed across aphid genetic clusters and suggested that bacterial endosymbionts affect population divergence and speciation^83^. More experiments are needed to uncover whether micro-ecological variables exist in host plant-associated *D. citri* populations.

### Demographic history of populations

Both neutrality tests and unimodal mismatch distributions revealed an expansion of *D. citri* populations. The recent population expansion of *D. citri* and its increasing damage around the world may have resulted from the recent sharp expansion of citrus varieties and growing area, which has provided more food options and ecological environments for *D. citri*^84^. Beck & Reese^85^ proposed that insect survivorship, fecundity, growth rate and activity can be affected by the quantity and quality of hosts. An abundance of hosts in a habitat will increase survival and fecundity and reduce mortality. As Murraya paniculata is the primary wild host for *D. citri*, the increase in population size of this species would distinctly improve the fecundity and survival capacity of the insect^13,86^, and the extensive commercial cultivation of citrus trees in recent years may also have contributed to an expansion of the insect’s territory^84^. Additionally, the warmer temperatures and increased precipitation not only contribute to *D. citri*’s physiological activity but also provide suitable living conditions for *D. citri* in relatively dry seasons in South China, increasing the number of habitats in Mainland China^6,87^.

## Materials and Methods

### Sample collection

**Figure 7:**
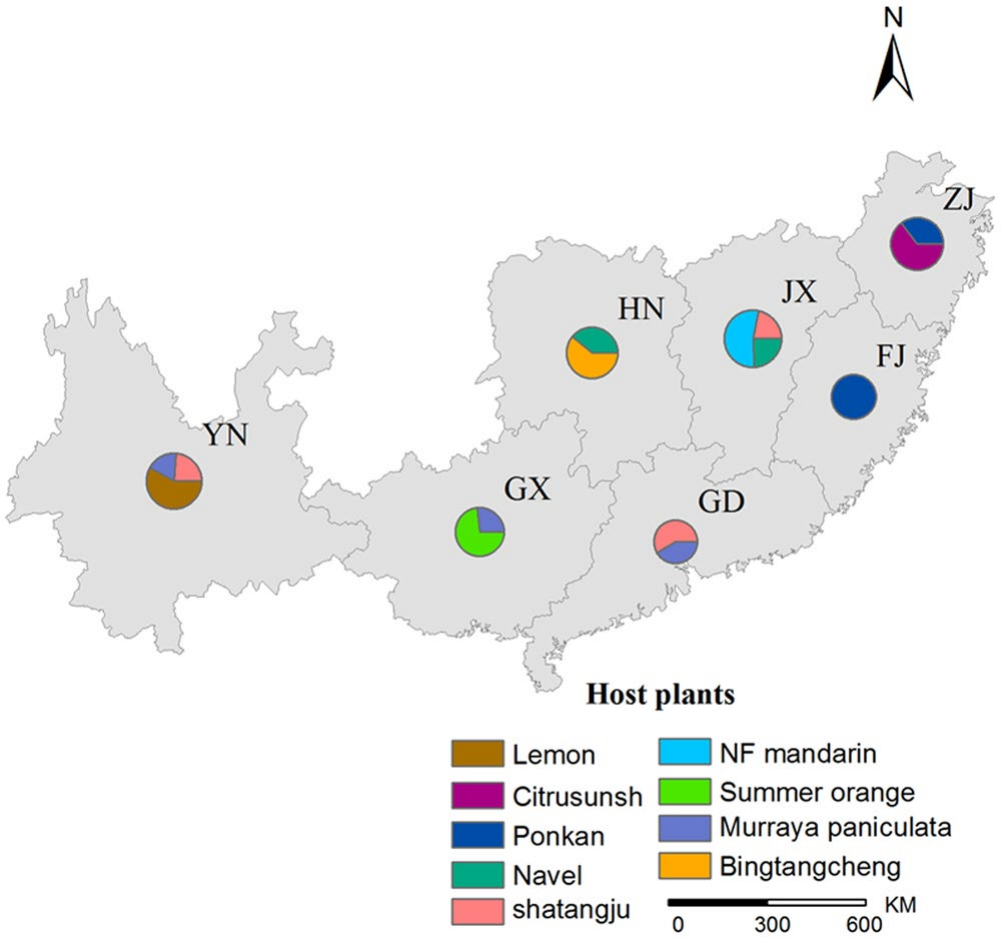
Geographical sites and host plants of sampled *D. citri* in China. The map was drawn with Arcgis 10.2 software (http://www.esri.com/arcgis/about-arcgis).

A total of 225 adult *D. citri* individuals were collected from 9 host plant types located in 20 citrus production regions across 7 governmental provinces in China (Fig. 7 and Table 6). Upon sampling, geographical coordinates and host plant species were recorded. To maximize the coverage of the genetic variation among samples, we collected *D. citri* from an additional 10 host plants within a 10-mile zone centered on each citrus sampling site per region. Samples were stored in 70% ethanol at 4 °C until DNA extraction.

**Table 6:** Details of *D. citri* samples used in the study.

Province	Location	Number	Geographical coordinates	Host plant
Zhejiang (ZJ)	Lishui	12	28°26′N 119°54′E	Ponkan
	Taizhou	22	28°38′N 121°09′E	Citrus unshiu
Hunan (HN)	Chenzhou	7	25°22′N 112°53′E	Navel
	Yongzhou	6	25°37′N 111°31′E	Navel
	Chenzhou	20	26°30′N 113°30′E	Bingtangcheng
Jiangxi (JX)	Fuzhou	22	27°12′N 116°30′E	NF mandarin
	Ganzhou	10	25°50′N 114°55′E	Navel
	Ganzhou	9	25°17′N 114°57′E	Shatangju
Fujian (FJ)	Quanzhou	25	25°23′N 118°16′E	Ponkan
Yunnan (YN)	Baoshan	22	24°58′N 98°52′E	Lemon
	Mangshi	9	24°26′N 98°26′E	Shatangju
	Ruili	7	24°3′N 97°56′E	Murraya paniculata
Guangxi (GX)	Guilin	4	25°16′N 110°19′E	Murraya paniculata
	Liuzhou	4	24°39′N 109°14′E	Murraya paniculata
	Guilin	22	24°24′N 110°23′E	Summer orange
Guangdong (GD)	Zhaoqing	10	23°8′N 112°5′E	Murraya paniculata
	Zhaoqing	7	23°2′N 112°23′E	Shatangju
	Guangzhou	7	23°9′N 113°20′E	Shatangju
Total		225		9

### DNA extraction and PCR amplification

Total genomic DNA was extracted from a single intact insect following a modified salt-extraction protocol^88^. The DNA was eluted in 40 mL TE buffer and stored at −20 °C. Final concentrations and 280/260, 260/230 ratios were estimated with a NanoDrop UV spectrophotometer (Techno Scientific, Wilmington, DE, USA). DNA amount per sample was normalized to 30 ng/μL. The polymerase chain reaction (PCR) protocols for SSR markers and mtDNA genes were as follows:

### SSR amplification and genotyping

Based on the entire genome, SSR Hunter 1.3 software^89^ was employed for SSR content. Primer pairs were designed in Software PRIMER v 3.0^90^ with sufficient flanking sequence and tested for amplification and polymorphism using DNA obtained from thirty individuals. The set of individuals was the same as those used for variability testing (see below) and individuals were rotated during initial screening such that a different set of thirty was used for each primer pair. The variability of the selected polymorphic loci was assessed in 225 specimens, and they were deposited in GenBank with the Accession nos.KY053427-KY053435. The conditions and characteristics of the SSR loci are provided in Table 1. For SSR genotyping, all forward primers were tagged with M13 (−21) (5′-TGTAAAACGACGGCCAGT-3′) at the 5′end. PCR amplifications were performed in a 10 μL reaction volume (4.5 μL Takara Master Mix [Shanghai Corporation], 0.4 μM of both forward and reverse primers, 0.2 μM dye labeled primer, 0.1% Triton X-100, 1.2 μL water and 1 μL DNA template [~30ng]). The PCR conditions were: initial denaturing at 95 °C for 2 min, followed by 35 cycles at 95 °C for 30 s, annealing at primer specific temperature for 45 s, extension at 72 °C for 45 s. Then, the reaction was paused at 72 °C, and fluorescently labeled (6- FAM, HEX or TAMRA) M13 primer was added. Amplification was continued for the next 10 cycles according to De Arruda^91^ with 30 min of final extension at 72 °C. Fragment analyses were run on an ABI 3730 DNA sequencer (Applied Biosystems) and analyzed with GENEMARKER 2.2.0 (Soft Genetics) using GS-500 (LIZ) as a size standard.

Sequencing of SSR loci was performed for the presence of size homoplasy detection. To isolate the specific bands of SSRs, the PCR products were separated on a 2.5% agarose gel. Only the best amplified bands for each SSR primer combination was collected and purified with QIA quick PCR Purification Kit (Omega). Those DNA fragments were sequenced with the specific forward or reverse SSR primer on an ABI3700 DNA analyser. The sequences obtained were checked for their homology with the SSR source sequence.

### Mitochondrial DNA amplification

Three mitochondrial genes (cytochrome c oxidase subunit 1, COI (781 bp); cytochrome b, Cytb (867 bp); and NADH dehydrogenase subunit 5, ND5 (750 bp)) were amplified and sequenced. Products were amplified by PCR in 25 µL reactions with 1.25 units of AmpliTaq DNA polymerase, 1 × PCR buffer, 2 mM of MgCl2, 0.4 mM of dNTPs and 0.5–1 µM of each primer. A detailed description of the primers used and PCR conditions can be found in the Supporting Information (Table S1). Sequencing was carried out using fluorescently labeled dideoxy terminators on an ABI 3130xl Genetic Analyzer (Applied Biosystems Corp.).

### Data analyses

#### SSR analysis, genetic diversity

We employed the Quantity One software (Biorad, USA) to estimate the band sizes adjusted for the standard molecular weight size marker (20 bp DNA ladder, TAKARA) and then formatted these size-based data to be used in GenAlEx v 6.0^92^ for further analysis. The genotypic data were used to estimate genetic diversity. Allele dropout and null alleles in the newly developed SSR data were checked using MICROCHECKER v 2.2.3^93^. The observed number of alleles (*N*_*A*_), observed heterozygosity (*Ho*), expected heterozygosity (*He*), percentage of polymorphic loci (*P*), Nei’s genetic diversity (Nei’s), and Nei’s original measure genetic distance (Nei’s *D*) were calculated with GenAlEx v 6.0 software. Deviations from Hardy-Weinberg equilibrium (HWE) and linkage equilibrium between loci were quantified in GENEPOP v 1.2^94^ within populations. The inbreeding coefficient (*F*_*IS*_) and confidence intervals were calculated in FSTAT v 1.2 with 1000 random permutations^95^.

#### Phylogenetic genealogy construction with SSRs

Unrooted neighbor-joining trees were constructed using matrices of chord genetic distance^96^ using the PHYLIP v3.5c^97^ software. The CONSENSE subroutine within PHYLIP was used to perform 1000 bootstraps of the distance matrix. The resulting trees were visualized using TREEVIEW v 1.6.6^98^.

#### Population structure based on SSRs

Different analyses were conducted to examine the contemporary population structure of *D. citri* populations in China. First, principal coordinate analysis (PCoA) was performed based on the simple matching coefficient using the decenter and eigenvector matrices in GenAlEx v 6.0 with 1000 random permutations. Second, analysis of molecular variance (AMOVA) was performed, where the total genetic variability was subdivided into three variance components, i.e., among clusters (F_CT_), among populations within clusters (F_SC_), within populations (F_ST_) using the software Arlequin v 3.5 with 1000 permutations^99,100^.

#### Genetic diversity and population structure with mtDNA

The 225 mtDNA sequences were compiled initially in Clustal-X^101^ and further refined with BioEdit 7.0^102^. Haplotype diversity (Hd) and nucleotide diversity (π) of total *D. citri* individuals were calculated as defined by Nei^103^ in DnaSP v 5.0^104^ with nonsyn and syn sites separately. Genetic parameters for each population grouped by either location or host, including number of haplotypes (Nh), haplotype diversity (Hd) and nucleotide diversity (π), and number of segregating sites were further analyzed with nonsyn sites in concatenated mtDNA. AMOVA analysis was performed in the same way as described for the SSR data analysis above to test for whether significant population structure exists using the HKY model^105^. Evidence of isolation by distance among sampling locations in China was obtained by examining the correlations between matrices of pairwise genetic distance (F_ST_) and pairwise physical distance^106^. Mantel tests^107^ were used to test the strength of the correlations between the matrices in GenALEx v 6.0.

#### Haplotype phylogeny with mtDNA

Substitution saturation was accessed for the alignments of single genes and concatenated dataset with DAMBE v. 5.3.46^37^. To measure the level of homoplasy, the consistency index (CI) was calculated using PAUP 4.0b10^108^. Phylogenetic analysis with the maximum parsimony (MP) method was carried out by PAUP* v4.0b10 using 100 replicates, MAXTREES = 200,000, random addition, TBR branch swapping, and MULTREES, with gaps treated as missing data. Support for the monophyletic groups revealed in the maximally parsimonious trees was examined using 1000 bootstrap replicates for the bootstrap (BP) consensus tree. In terms of syn and nonsyn substitutions per site of mtDNA, branch lengths of each mitochondrial gene were estimated with 26 previously defined sequences using a codon-based model of substitution within the codeml application in PAML v4.0^109^. Tree topologies were visualized using TREEVIEW v 1.6.6^98^. Further, we constructed a haplotype network with nonsyn sites in the concatenated mtDNA to visualize relationships among haplotypes in the program POPART based on a median-joining algorithm^110^ designed for populations of closely related samples allowing persistent ancestral nodes and multi-furcation in the network as extra information for population genetic structures.

#### Demographic history *D. citri* population in China

Signatures of *D. citri* populations’ demographic changes were investigated using three neutrality tests: Tajima’s *D*^111^, Fu’s *Fs*^112^, and Ramos-Onsins & Rozas’ R^2 ^^113^. All methods are premised on neutral selection. The neutral theory states that the majority of the genome is variable and neutral. Actually, this assumption is not true^22^. According to Huang *et al*.^36^, the value of the theory is its description for the linear phase, which has been retained by the MGD. Hence, the following neutrality tests of *D. citri* were from the nonsyn sites. The examination of deviation from neutrality by both *D* and *Fs* indices was based on 1000 coalescent simulations, as implemented in Arlequin v 3.5. Significant negative *D* and *Fs* values can be interpreted as signatures of population expansion. The R^2^ statistic of Ramos-Onsins& Rozas, which has more statistical power when population sizes are small, was calculated using a coalescent simulation algorithm implemented in DnaSP, with 1000 simulations.

To provide other estimates of population size changes, we also examined site mismatch distributions. Contrasting plots of observed and theoretical distributions of site differences yield insight into past population demographics. The expected mismatch distributions under a sudden expansion model were computed in Arlequinv 3.5. The sum of squared deviations (SSD) between observed and expected distributions was used as a measure of fit, and the probability (P) of obtaining a simulated SSD greater than or equal to the expected SSD was computed by randomization. If this probability (P) was >0.05, the expansion model was accepted. Graphical representation was implemented in Arlequin.

### Data Availability

GenBank accessions of nine polymorphic loci are KY053427-KY053435.

## Electronic supplementary material

Supplementary information
