# Supplementary material for: Population genetic structure of Diaphorina citri Kuwayama (Hemiptera: Liviidae): host-driven genetic differentiation in China
Source: Sci Rep. 2018 Jan 24;8:1473. doi: 10.1038/s41598-018-19533-5 (PMC5784137; doi:10.1038/s41598-018-19533-5)
Supplement: Supplementary file 1 — Supplementary information [file 41598_2018_19533_MOESM1_ESM.pdf]

Population genetic structure of *Diaphorina citri* Kuwayama (Hemiptera: Liviidae): host-driven genetic differentiation in China

Lixue Meng[1], Yongmo Wang[2], Wen-Hua Wei[3], Hongyu Zhang[1*]

[1] Key Laboratory of Horticultural Plant Biology (MOE), State Key Laboratory of Agricultural Microbiology, Institute of Urban and Horticultural Entomology, College of Plant Science and Technology, Huazhong Agricultural University, Wuhan 430070, China; [2] Hubei Insect Resources Utilization and Sustainable Pest Management Key Laboratory, College of Plant Science and Technology, Huazhong Agricultural University, Wuhan, Hubei, P.R. China; [3] Department of Women's and Children's Health, Dunedin School of Medicine, University of Otago, Dunedin 9016, New Zealand.

* E-mail: hongyu.zhang@mail.hzau.edu.cn

Supporting information

Table S1 Primers used for PCR and sequencing in the study.

	Primer	Sequence	PCR protocol
COI	COI-F<br>COI-R	5’- AGGAGGTGGAGACCCAATCT -3’<br>5’- TCAATTGGGGGAGAGTTTTG - 3’	94 ℃ for 2 min; 35 cycles of 94 ℃ for 30 s, 53 ℃ for 30 s, 72 ℃ for 1 min; 72 ℃ for 10 min. (Boykin *et al.* 2012)
Cytb	Cytb F<br>Cytb R	5’- GGTAAAGTTCCCCGAATAAC -3’<br>5’- AGCCAGGTTGGTTTCTATCT -3’	95 ℃ for 4 min; 40 cycles of 95 ℃ for 30 s, 59 ℃ for 30 s, 72 ℃ for1 min; 72 ℃ for 10 min.
ND5	ND5F<br>ND5R	5’- TTTTTGACCCAGCTACTCAT -3’<br>5’- GATTTTTAGTCCGAGCATTG -3’	94 ℃ for 5 min; 35 cycles of 94 ℃ for 30 s, 54 ℃ for 1 min, 72 ℃ for 90 s; 72 ℃ for 10 min.

COI

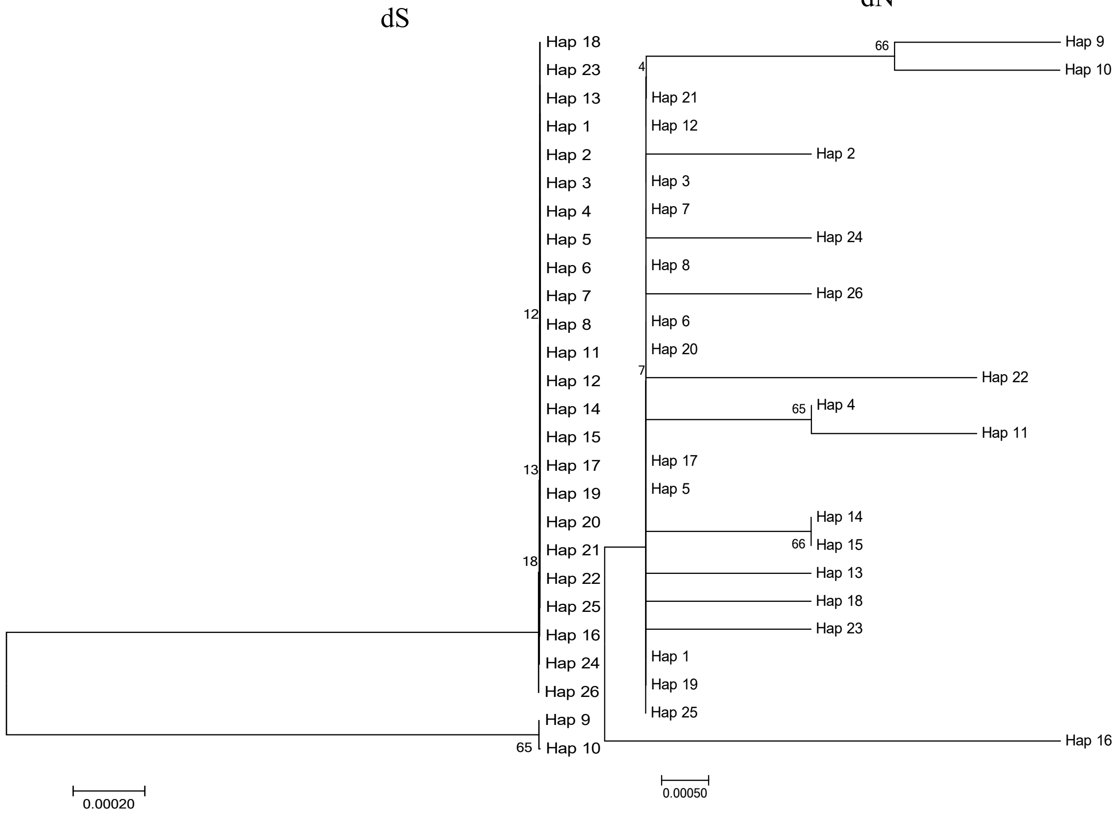
dS
dN
Hap 18
Hap 23
Hap 13
Hap 1
Hap 2
Hap 3
Hap 4
Hap 5
Hap 6
Hap 7
Hap 8
Hap 11
Hap 12
Hap 14
Hap 15
Hap 17
Hap 19
Hap 20
Hap 21
Hap 22
Hap 25
Hap 16
Hap 24
Hap 26
Hap 9
Hap 10
12
13
18
65
0.00020
66
4
Hap 9
Hap 10
Hap 21
Hap 12
Hap 2
Hap 3
Hap 7
Hap 24
Hap 8
Hap 26
Hap 6
Hap 20
7
Hap 22
65
Hap 4
Hap 11
Hap 17
Hap 5
Hap 14
66
Hap 15
Hap 13
Hap 18
Hap 23
Hap 1
Hap 19
Hap 25
Hap 16
0.00050

ND5

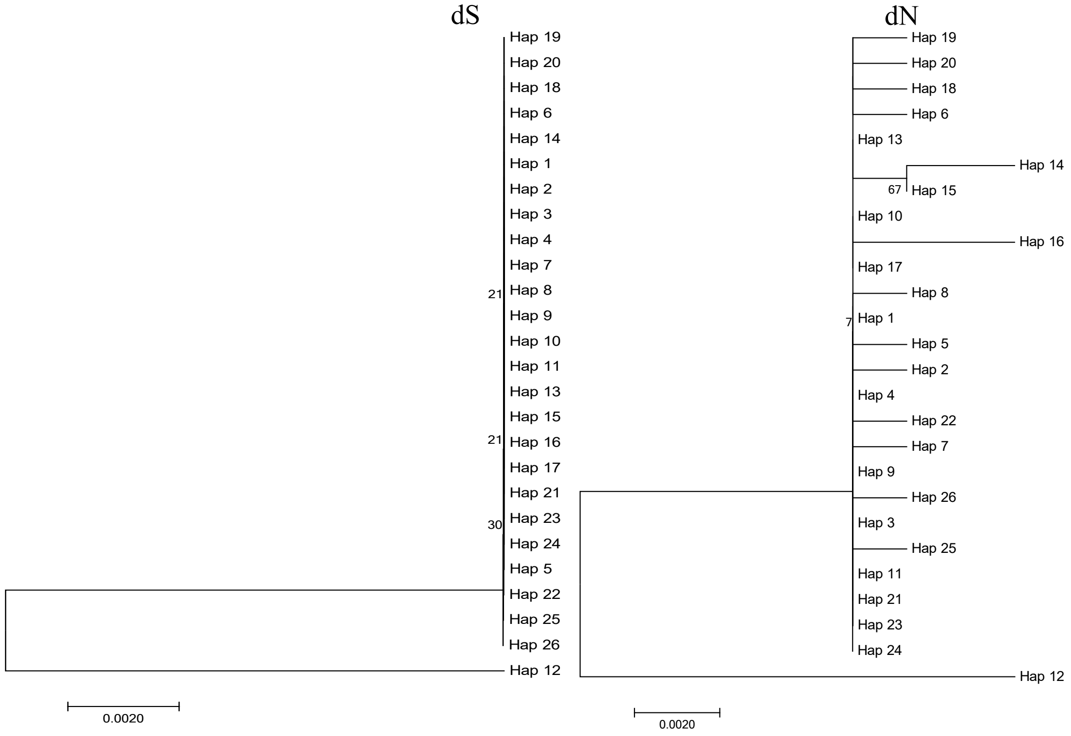
dS
dN
Hap 19
Hap 20
Hap 18
Hap 6
Hap 14
Hap 1
Hap 2
Hap 3
Hap 4
Hap 7
Hap 8
Hap 9
Hap 10
Hap 11
Hap 13
Hap 15
Hap 16
Hap 17
Hap 21
Hap 23
Hap 24
Hap 5
Hap 22
Hap 25
Hap 26
Hap 12
21
21
30
0.0020
Hap 19
Hap 20
Hap 18
Hap 6
Hap 13
Hap 14
67
Hap 15
Hap 10
Hap 16
Hap 17
Hap 8
7
Hap 1
Hap 5
Hap 2
Hap 4
Hap 22
Hap 7
Hap 9
Hap 26
Hap 3
Hap 25
Hap 11
Hap 21
Hap 23
Hap 24
Hap 12
0.0020

Cytb

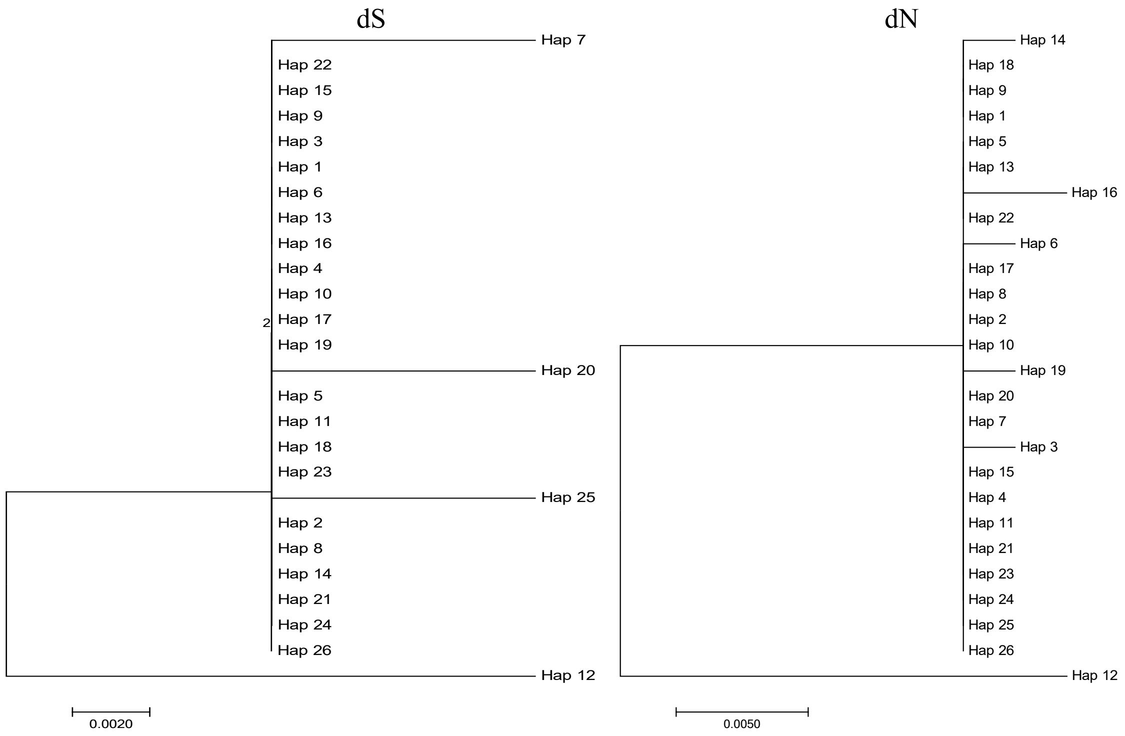

Concatenated gene

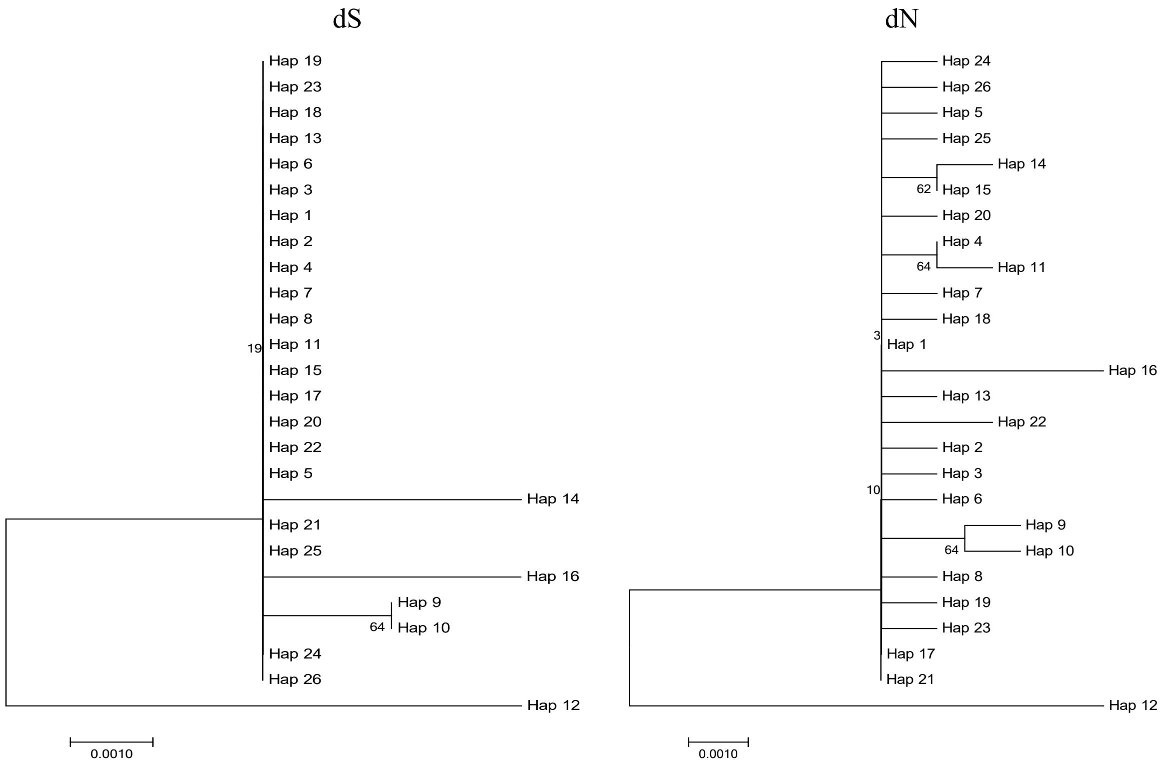

Fig. S1 dN and dS trees for mitochondrial genes. Branch lengths are in terms of synonymous (dS) or non-synonymous (dN) substitutions per site as estimated by PAML under a constrained topology.

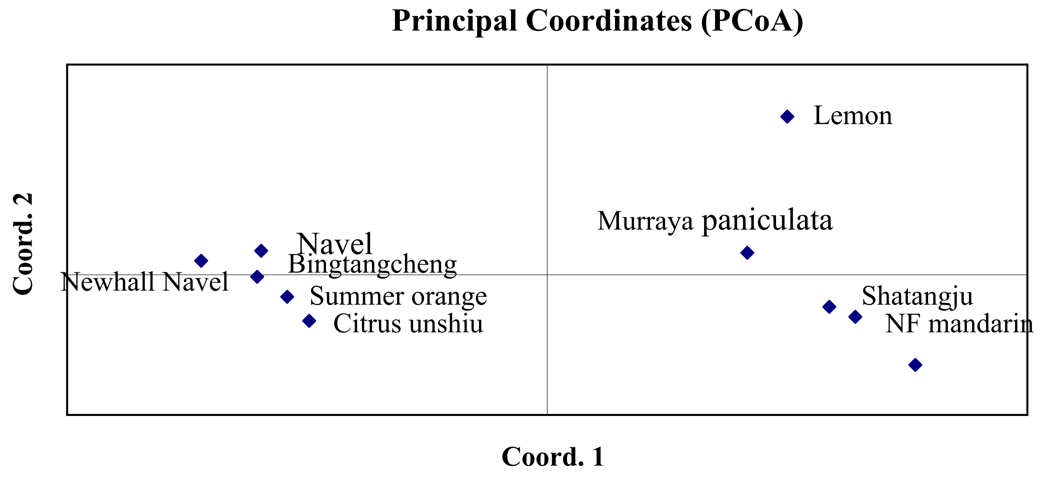

Fig. S2 Principal coordinate analysis (PCoA) of pairwise distances between populations of *D. citri* with SSR markers.

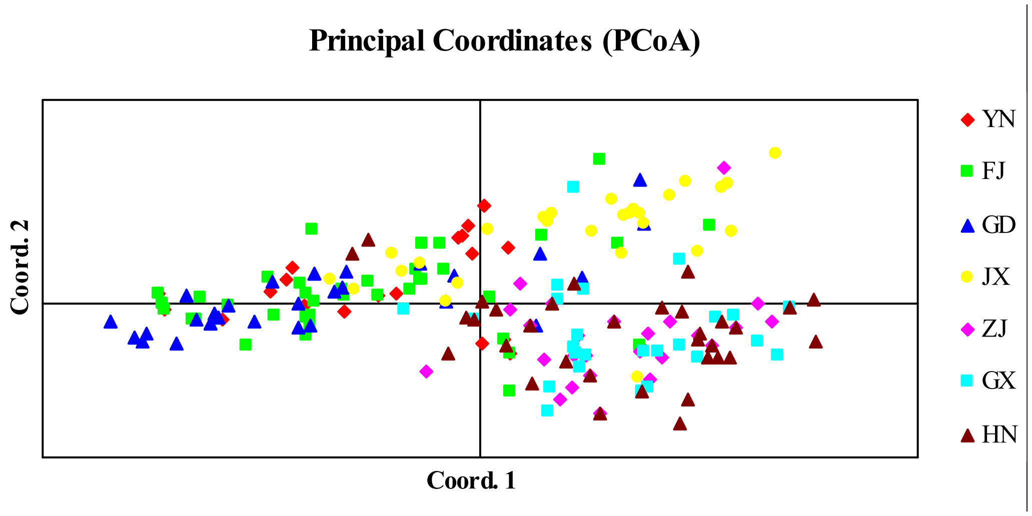

Fig. S3 Principal coordinate analysis (PCoA) of pairwise distances between individuals of *D. citri* with SSR markers. Geographical populations are indicated by different colors.

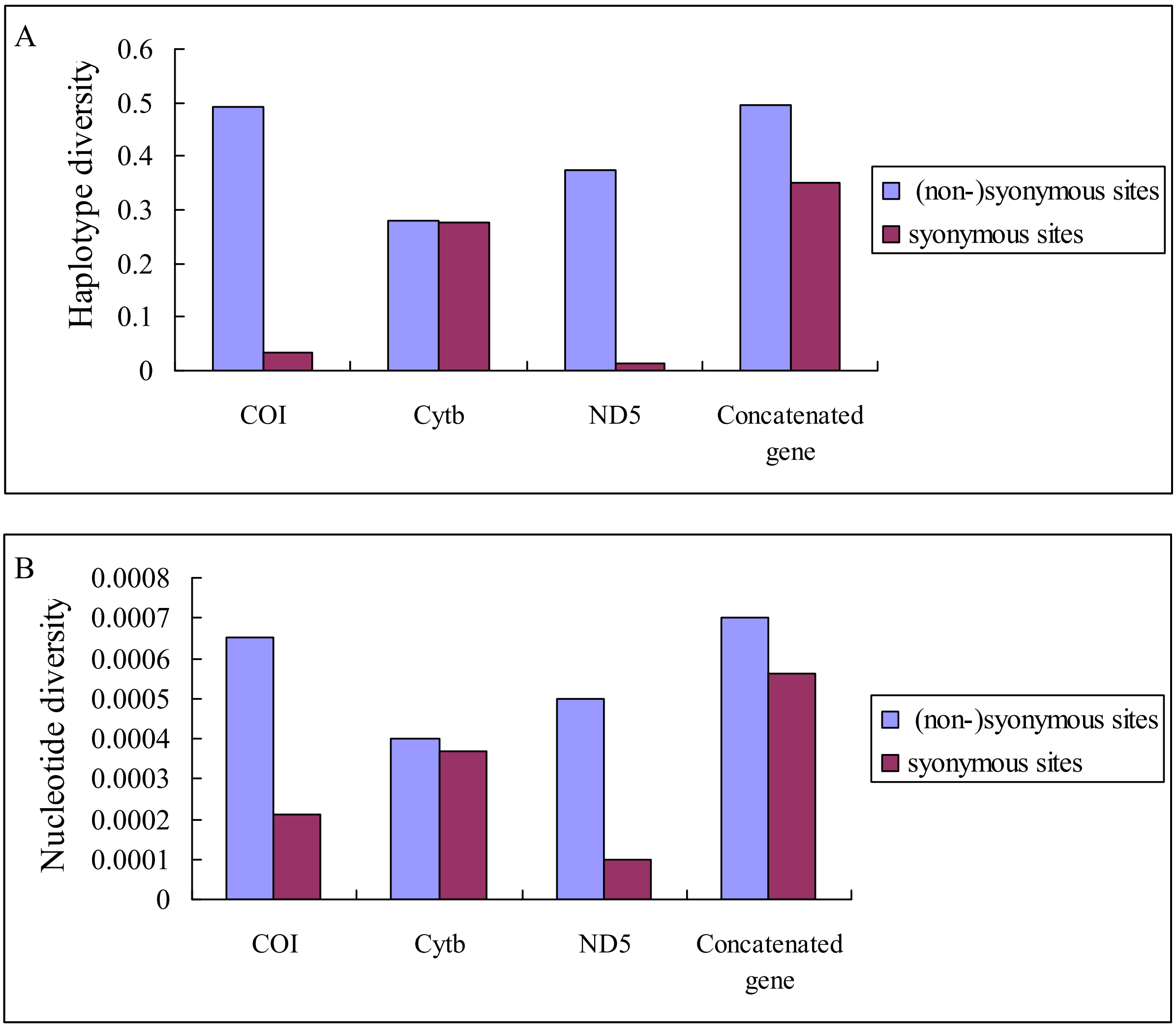

Fig. S4 Hplotype diversity (A), nucleotide diversity (B) calculated with syn sites and nonsyn sites in mtDNA.
